# Imported case of Middle East respiratory syndrome coronavirus (MERS-CoV) infection from Oman to Thailand, June 2015

**DOI:** 10.2807/1560-7917.ES.2017.22.33.30598

**Published:** 2017-08-17

**Authors:** Tanarak Plipat, Rome Buathong, Supaporn Wacharapluesadee, Potjaman Siriarayapon, Chakrarat Pittayawonganon, Chariya Sangsajja, Thongchai Kaewpom, Sininat Petcharat, Teerada Ponpinit, Jaruphan Jumpasri, Yutthana Joyjinda, Apaporn Rodpan, Siriporn Ghai, Akanitt Jittmittraphap, Sarawut Khongwichit, Duncan R Smith, Victor M Corman, Christian Drosten, Thiravat Hemachudha

**Affiliations:** 1 Bureau of Epidemiology, Department of Disease Control, Ministry of Public Health, Nonthaburi, Thailand; 2 World Health Organization Collaborating Centre for Research and Training on Viral Zoonoses, King Chulalongkorn Memorial Hospital, Faculty of Medicine, Chulalongkorn University, Bangkok, Thailand; 3 Bamrasnaradura Infectious Diseases Institute, Department of Disease Control, Ministry of Public Health, Nonthaburi, Thailand; 4 Department of Microbiology and Immunology, Faculty of Tropical Medicine Mahidol University, Bangkok, Thailand; 5 Institute of Molecular Biosciences, Mahidol University, Bangkok, Thailand; 6 Institute of Virology, University of Bonn Medical Centre, Bonn, Germany; 7 German Centre for Infection Research, Partner Site Bonn-Cologne, Bonn, Germany

**Keywords:** MERS-CoV, MERS, Thailand, imported case, diagnostic, contact tracing

## Abstract

Thailand reported the first Middle East respiratory syndrome (MERS) case on 18 June 2015 (day 4) in an Omani patient with heart condition who was diagnosed with pneumonia on hospital admission on 15 June 2015 (day 1). Two false negative RT-PCR on upper respiratory tract samples on days 2 and 3 led to a 48-hour diagnosis delay and a decision to transfer the patient out of the negative pressure unit (NPU). Subsequent examination of sputum later on day 3 confirmed MERS coronavirus (MERS-CoV) infection. The patient was immediately moved back into the NPU and then transferred to Bamrasnaradura Infectious Disease Institute. Over 170 contacts were traced; 48 were quarantined and 122 self-monitored for symptoms. High-risk close contacts exhibiting no symptoms, and whose laboratory testing on the 12th day after exposure was negative, were released on the 14th day. The Omani Ministry of Health (MOH) was immediately notified using the International Health Regulation (IHR) mechanism. Outbreak investigation was conducted in Oman, and was both published on the World Health Organization (WHO) intranet and shared with Thailand’s IHR focal point. The key to successful infection control, with no secondary transmission, were the collaborative efforts among hospitals, laboratories and MOHs of both countries.

## Introduction

### Background

From 2012 to 21 July 2017, there have been 2,040 reported laboratory-confirmed cases and 712 deaths from Middle East respiratory syndrome coronavirus (MERS-CoV) infection in 27 countries [1]. A single imported case of Middle East respiratory syndrome (MERS) in South Korea, identified on 20 May 2015, resulted in 150 laboratory-confirmed cases, amplified by infection in hospitals and the transfer of patients within and between hospitals, and caused 15 deaths within 26 days, mainly among patients, visitors and healthcare personnel [2]. This highlighted the need for vigilant surveillance and the importance of swift and thorough contact tracing.

The Thai Ministry of Public Health (MOPH) launched MERS surveillance and made MERS a notifiable disease in 2012, particularly targeting people travelling into Thailand from affected countries. It also initiated a nationwide public education campaign [3]. In 2015, MERS-CoV infection was classified as a dangerous communicable disease in Thailand according to the Communicable Act B.E. 2523 (AD 1980). Being added to this Act required all probable and confirmed cases and their close contacts to be quarantined in a designated area for the duration of the maximum incubation period of 14 days [4].

There have been increasing numbers of incoming travellers from the Middle East seeking medical care in Thailand in the past decade. More than 1.3 million medical tourists travelled to Thailand in 2015, of which 14.2% were from United Arab Emirates (UAE) and Oman [5]. Despite that, only three cases of MERS have been confirmed as of July 2017 [6].

This study shows that, in addition to needing collaboration among different organisations during an outbreak, diagnosis cannot rely only on laboratory examination alone, especially when the specimen was not suitable. A negative laboratory result in a patient from an endemic region with MERS-like clinical signs still demands cautious infection control measures in an isolation unit.

### The event

On 15 June 2015 (day 1), A 75-year old Omani man travelled to Thailand, seeking treatment for his heart condition. Upon arriving at the airport, the patient took a taxi to a hotel and checked in before leaving to a private hospital in another taxi. Upon presentation at the emergency room at the private hospital, the patient was promptly diagnosed with heart failure and possible pneumonia. As the patient had travelled from the Middle East that day, MERS was suspected and he was isolated in a negative pressure room (NPU). On day 3, the private hospital notified the Bureau of Epidemiology, under the Thai MOPH, of the Omani patient. RT-PCR for MERS-CoV on upper respiratory tract samples (nasopharyngeal swabs) that were sent on days 2 and 3 resulted in false negatives, leading to a 48-hour delay in diagnosis and a decision to transfer the patient out of the negative pressure unit (NPU) on day 3. Subsequent examination of a sputum sample later on day 3 confirmed MERS-CoV infection in the patient. The patient was immediately transferred back into the NPU. On day 4, Thailand’s MOPH officially reported the first imported MERS case and the patient was transferred from the private hospital to Bamrasnaradura Infectious Disease Institute (BIDI).

The Omani Ministry of Health (MOH) was immediately notified as per the International Health Regulation (IHR) mechanism (day 4). Outbreak investigation was conducted in Oman, and the Oman IHR focal point published the results of this investigation on the World Health Organization (WHO)’s intranet to which all IHR focal points worldwide, including the one in Thailand, have access. The following report provides a brief patient history and clinical report, detailed laboratory findings and the diagnostic challenges faced in the first imported case of MERS in Thailand.

## Methods

Over 170 individuals, including 48 with high-risk of exposure were traced. Thirty-six high-risk close contacts were quarantined in Thailand and 40 low-risk contacts were monitored in Oman. Another 12 high-risk close contacts (airline crew members) were quarantined in the country they were situated when traced. Medical records from the private hospital and BIDI under the Department of Disease Control, under the Thai MOPH, were reviewed [7]. Further, the patient and their family members were asked to elaborate on the clinical presentations and previous medical care in Oman by Thai investigators. Information from the Omani MOH was obtained via the IHR mechanism during investigation.

### Laboratory investigation: PCR assay

In accordance with WHO interim guidelines for laboratory testing for MERS-CoV [8], MERS-CoV RNA was tested in sputum (pre-treated with *N*-acetylcysteine) and via nasopharyngeal swab (when sputum was not available), using QIAamp viral RNA mini kit (Qiagen, Hilden, Germany) for extraction. Two real-time RT-PCR assays targeting upstream of envelope (UpE) and open reading frame 1a (ORF-1a) genes [9,10], and one RT-PCR assay for generating amplicons for sequencing, targeting the betacoronavirus RNA-dependent RNA polymerase (RdRp) gene (RdRpSeq assay) [10], were performed simultaneously by WHO Collaborating Centre for Research and Training on Viral Zoonoses, Faculty of Medicine, Chulalongkorn University (WHOCC) to increase efficiency and allow reporting of results within 24 hours of receiving the samples.

### Case monitoring

Respiratory specimens (sputum, nasopharyngeal and throat swabs) were collected daily from the index case from the time of patient isolation on day 5 through to day 17. The respiratory samples were sent to three centres, the BIDI, the Thai National Institute of Health (NIH) and WHOCC, for parallel real-time PCR testing of MERS-CoV. Additional molecular sequencing was performed by WHOCC.

### Whole genome sequencing and phylogenetic analyses

The whole genome amplification of MERS-CoV was carried out from extracted viral RNA from collected sputum of the index case. Seventy sets of specific primer pairs were used to amplify the complete genome as previously described [11], followed by Sanger sequencing.

For the analysis, all MERS-CoV genomes with complete coding sequences available in GenBank as of 30 December 2016 (n = 233), were compared with the MERS-CoV genome obtained in this study. Sequences showing less than 35 divergent nt positions and two representatives of the five lineages defined by Sabir et al. [12], were selected and used for phylogenetic analysis. A phylogenetic tree was constructed using the maximum likelihood method based on the general time reversible model and 1,000 bootstrap replicates in MEGA7 [13].

### Contact tracing, active case finding, quarantine and isolation

Contact tracing was immediately implemented by the Thai MOPH. Contacts were divided into two categories; high-risk and low-risk. A high-risk close-contact was defined as any person who was within 1 m of contact with the index case while the patient was symptomatic, regardless of duration of contact. Airline passengers seated in the two rows surrounding the index case’s seat were also considered high-risk close contacts as per WHO guidelines [14]. A low-risk contact was any person who had been in contact with the patient while the patient was symptomatic, but from a distance of more than 1 m. People were considered non-contacts if there was no evidence of direct contact with the patient or if they were not likely to be in contact with respiratory droplets, the means of transmission for MERS-CoV.

Several methods of contact tracing and active case finding were applied depending on the nature of contact, contact location, degree of symptoms at the time of contact, etc. At the hospital, attending physicians’ and nurses’ contact status was determined via interview and the hospital surveillance camera. At the hotel, potential contacts were identified by interviewing the personnel on-duty when the patient checked in and by using the hotel’s surveillance camera. The investigation team from the Department of Disease Control (DDC) at the Thai MOPH identified the airport-to-hotel taxi driver using the airport taxi booking slip and the hotel-to-hospital taxi driver by looking at the surveillance camera from the Traffic Control Department. The airline provided the investigation team with the passenger manifest and the Thai authorities identified passengers’ local address using immigration arrival cards. Local health authorities in relevant provinces were informed and asked by the investigation team to locate and contact the identified passengers in their jurisdiction. Some passengers voluntarily reported to a hospital or health authority in response to the MOPH’s announcement of first imported MERS case in Thailand. Central authorities were responsible for locating all high-risk close-contacts, while local authorities were responsible for low-risk contacts. The time lapse between the affirmed diagnosis and each contact-tracing varied. Contacts at the hospital were identified within a day, while other high-risk close contacts, such as passengers on the flight, were identified within 3 days. Other low-risk contacts were traced within 7 days.

Patients who were in the same NPU ward as the index case at the private hospital on day 1 were monitored despite being considered non-contacts because: i) the room for each patient was separated, ii) they had no direct contact with the index case and iii) the known mode of transmission of MERS-CoV is respiratory droplet. Further, patients in the ICU, which is where the index case was moved after being taken out of the NPU 8 hours before diagnosis on day 3, were monitored, despite being non-contacts. In the event they developed a new episode of fever or respiratory symptoms, samples were collected and sent for testing to rule out MERS-CoV infection. Another concern was ICU healthcare workers’ simultaneous care of several patients. Prompt quarantine and monitoring of patients in the ICU was to be implemented if any ICU healthcare worker developed any symptoms or was diagnosed with MERS-CoV infection.

Most high-risk close contacts were quarantined and all were continuously monitored for 14 days. Nasopharyngeal and throat swabs, stored in single viral transport media, from 36 high-risk close contacts were collected on two occasions as per the Bureau of Epidemiology guidelines [15]: first upon identification as being a high-risk close contact and second on day 12 during the quarantine period. Specimens were duplicated and sent to any two of three laboratories (BIDI, NIH and WHOCC) for parallel real-time PCR testing of MERS-CoV, using both WHO and commercial primers for any given sample. In line with the Thai Communicable Disease Act, high-risk close contacts were only released after completing 14 days of quarantine and if laboratory testing on the 12th day of quarantine was negative.

Sera from three high-risk close contacts (the close relatives who travelled with the patient to Thailand) were sent to the Institute of Virology, University of Bonn Medical Centre, Bonn, Germany to test for anti-MERS IgG and IgM using MERS-CoV infected Vero cells for immunofluorescence assay (Anti-MERS-CoV IIFT, EUROIMMUN, Lübeck, Germany).

## Results

### Laboratory diagnosis and clinical picture

The real-time RT-PCR results on the patient’s nasopharyngeal swabs were negative for UpE and ORF-1a gene targets on days 2 and 3 (Table 1), and the patient was thus transferred to a non-NPU in the ICU. However, the third sample from sputum that was taken later on day 3 as the patient’s condition deteriorated and that underwent three simultaneous RT-PCR assays at the WHOCC, was positive for UpE, ORF-1a and RdRp gene targets. When WHOCC confirmed sputum was positive for MERS-CoV infection, the patient was immediately transferred back to the NPU that night. MERS-CoV was confirmed via sequencing within 24 hours by WHOCC.

**Table 1:** RT-PCR results for the first imported case of MERS, Thailand, June 2015

Date	Day	Specimen type	Real-time RT-PCR (Ct)		RT-PCR and partial sequencing
			UpE gene	ORF-1a gene	RdRp gene
16 Jun 2015	2	Nasopharyngeal swab^a^	ND	ND	NA
17 Jun 2015 (AM)	3	Nasopharyngeal swab^a^	ND	ND	NA
**17 Jun 2015 (PM)**	**3**	**Sputum**	**Detected** **(33.75)**	**Detected** **(34.23)**	**Positive^b^**
**18 Jun 2015**	**4**	**Sputum**	**Detected** **(30.97)**	**Detected** **(30.55)**	**Positive^b^**
20 Jun 2015	6	Sputum and nasopharyngeal swab	ND	ND	ND
21 Jun 2015	7	Nasopharyngeal swab^a^	ND	ND	ND
22 Jun 2015	8	Nasopharyngeal swab^a^	ND	ND	ND
1 July 2015	17	Nasopharyngeal swab^a^	ND	ND	ND

Ct: cycle threshold; MERS: Middle East respiratory syndrome; NA: not available; ND: not detected; ORF-1a: open reading frame 1a; RdRp: RNA-dependent RNA polymerase; UpE: upstream of envelope.

^a^ Sputum not available.

^b^ Partial sequencing of 185 nt of RdRp gene showed 99% (185/186 bp) identity to the 2015 MERS-CoV isolate from a human case in Riyadh (GenBank accession number KT026454).

The patient was referred to and isolated at BIDI on the morning day 4. The patient’s clinical presentation at that time was diffused bilateral pneumonia with pending acute respiratory distress syndrome [7]. He did not report any previous illnesses pertaining to these symptoms.

Later the same day, sputum was collected from the patient for reconfirmation, which tested positive for UpE and ORF-1a genes by four different laboratories: the NIH, Ramathibodi Hospital, BIDI and WHOCC. The Thai MOPH proceeded to publicly announce the first confirmed imported MERS case in the evening of 18 June 2015 (day 4). The patient was monitored for MERS until 1 July 2015 (day 17) and was discharged on 3 July 2015 (day 19).

### Retrospective case history

Upon laboratory confirmation on day 3, the case was immediately notified to the WHO. In order to support local handling of the outbreak, an epidemiologist and a risk communication expert were deployed from the WHO South-East Asia Regional Office and WHO Headquarters, respectively.

**Figure 1:**
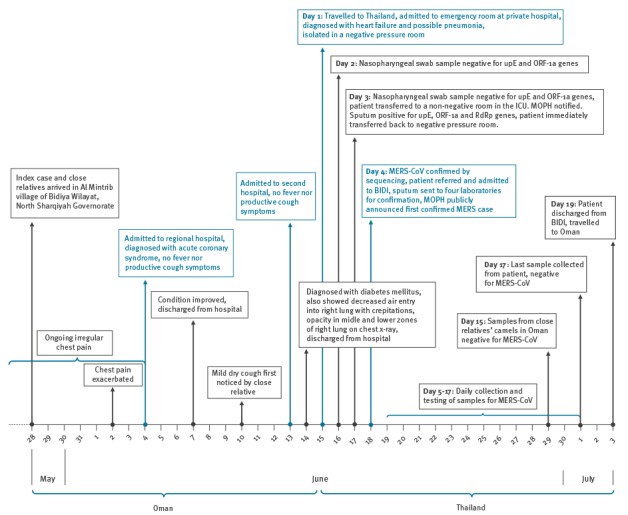
Timeline of events for the first imported case of MERS in Thailand, Thailand and Oman, May–July 2015

BIDI: Bamrasnaradura Infectious Disease Institute; MERS: Middle East respiratory syndrome; MERS-CoV: Middle East respiratory syndrome coronavirus; MOPH: Thai Ministry of Public Health; ORF-1a: open reading frame 1a; RdRp: RNA-dependent RNA polymerase; UpE: upstream of envelope.

The epidemiological investigation revealed retrospectively that on 4 June 2015, 11 days before the admission to the private hospital in Thailand, the patient was admitted to a regional hospital in Oman, with retrosternal and left-sided chest pain, which was radiating to his left arm (Figure 1). The condition was associated with shortness of breath on exertion and was considered typical cardiac pain. The patient was diagnosed with acute coronary syndrome. Three days later, on 7 June 2015, his condition had improved and he was discharged. A close relative of the patient observed a dry cough of mild degree in the patient since 10 June 2015. On 13 June 2015, he was admitted to a second hospital, displaying signs of somnolence, fatigue and elevated blood sugar level; he was diagnosed with diabetes mellitus on 14 June 2015. There was also decreased air entry in the right lung with fine crepitations. Chest X-ray showed opacity in middle and lower zones of right lung. Both hospitals’ medical records confirmed the patient did not exhibit any fever or cough symptoms. The patient was discharged that day with a follow-up appointment at a regional hospital scheduled for 16 June 2015; however, the patient wished to seek medical care in Thailand and flew there on 15 June 2016. 

### Phylogenetic analysis

**Figure 2:**
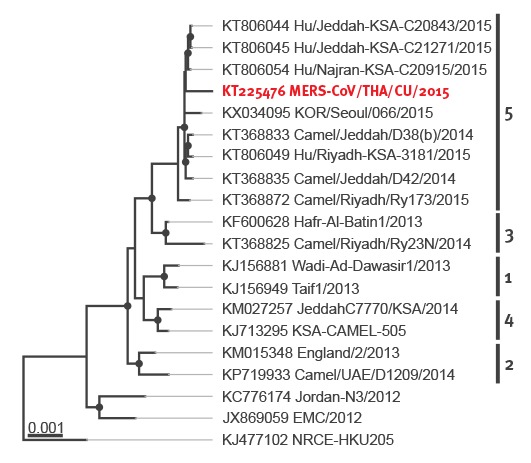
Maximum-likelihood phylogeny of representative MERS-CoV genomes and the complete MERS-CoV genome obtained for this study, first imported case of MERS in Thailand, June 2015

The virus sequence (THA/CU/2015) obtained from the patient was submitted to GenBank (GenBank accession number KT225476). THA/CU/2015 showed closest relations (99.91% nt identity) to three human MERS-CoV strains isolated in Saudi Arabia in 2015 (GenBank accession numbers KT806044, KT806045 and KT806054). Figure 2 shows the phylogenetic tree constructed from the MERS-CoV whole genome obtained from the patient (THA/CU/2015, 29,809 bp), among the closest relatives and representatives for each MERS-CoV lineage defined by Sabir et al. [12].

MERS-CoV: Middle East respiratory syndrome coronavirus.

Filled circles at nodes indicate bootstrap supports above 75% (1,000 replicates). The virus sequence obtained from the case described in this manuscript (THA/CU/2015, GenBank accession number KT225476) is shown in red. The trees were rooted by Camel/Egypt/NRCE-HKU205 (GenBank accession number KJ477102). MERS-CoV lineages defined by Sabir et al. [12] are given on the right.

### Exposure history in Oman

Correspondences with a close relative of the patient and the Omani MOH revealed that the patient was a fisherman from Ghasil village, South Sharqiya Governorate, but spent June–August in Al Mintrib village of Bidiya Wilayat, North Sharqiyah Governorate. The patient neither had any history of travel outside Oman nor contact with anyone with a history of travel outside Oman within the 14 days preceding his travel to Thailand. Further, the patient had no contact with any person with acute respiratory infection or confirmed MERS before developing symptoms. The family used to own one camel but had not had contact with that camel for few months. A close relative who lived near the patient, but did not travel to Thailand with the patient, owned and cared for three camels. Samples collected from these camels tested negative in Oman for MERS-CoV by RT-PCR on day 15. It is also noteworthy that the patient and the patient’s close relatives never consumed raw camel milk or camel urine.

### Contact tracing and active case finding

A total of 211 contacts of the index case were identified after the patient was confirmed to have MERS. In Thailand, 170 contacts (excluding the 42 healthcare personnel at BIDI, investigated separately in the report by Wiboonchutikul et al. [7]) were identified. Of these 170, 48 were high-risk close contacts and 122 were low-risk contacts (Table 2). All patients treated in the same ward (ICU) at the private hospital of first admission of index case before MERS diagnosis were identified as non-contact, however they were fully monitored and followed up for 14 days, as per the Thai MOPH’s protocol, as a precautionary measure. In Oman, the outbreak investigation determined there to be 41 low-risk contacts and this information was published on the dedicated WHO system. Fortunately, there was no secondary transmission associated with this case.

**Table 2:** Tracing contacts of the first imported case of MERS in Thailand, Thailand and Oman, June 2015 (n = 211)

Type of contact	Number of contacts	Course of action
**High-risk close contacts in Thailand (n = 48)**		
Close relatives travelling with the patient	3	Quarantined. Laboratory testing results for MERS-CoV were negative.
Airline passengers who sat in the two rows around the patient	14^a^	Quarantined. Laboratory testing results for MERS-CoV were negative.
Airline crew members	12	Identified, but left Thailand for their return flight on 15 June 2015 (day 1). Self-quarantined and self-monitored for symptoms, being off-service for the duration of the quarantine period. None reported to have developed any illness.
Healthcare workers at the private hospital	17	All were immediately quarantined in the hospital. Their laboratory testing were negative for MERS-CoV.
Taxi drivers	2	Quarantined. Laboratory testing for MERS-CoV were negative.
**Low-risk contacts in Thailand (n = 122)**		
Airline passengers	63	Self-monitored for symptoms, with social distancing.
Hotel staff	6	
Healthcare workers	53	
**Low-risk contacts in Oman (n = 41)**		
Close relatives	11	Assigned for two weeks of follow-up from the last date of exposure. One close-contact was assigned to be tested for MERS-CoV infection, but refused to cooperate.
Healthcare contacts at the first hospital	20	
Healthcare contacts at the second hospital	10	

MERS: Middle East respiratory syndrome; MERS-CoV: Middle East respiratory syndrome coronavirus.

^a^ Only one of whom was a Thai national.

### High-risk close contacts

Three of the patient’s close relatives who (45, 30 and 25 years of age) travelled with the patient to Thailand and took care of the patient. They were also isolated at BIDI on the morning of 18 June 2015 (day 4) along with the patient. Their RT-PCR tests (each person tested four times) were negative for MERS-CoV. However, they were closely monitored for symptoms until 1 July 2015 (day 17). Sera from these three close relatives collected on 19 June and 1 July 2015 (days 5 and 17), were sent to the Institute of Virology at University of Bonn Medical Centre, Bonn, Germany, and all tested negative for anti-MERS IgG and IgM using MERS-CoV infected Vero cells for immunofluorescence assay (Anti-MERS-CoV IIFT, EUROIMMUN, Lübeck, Germany) (Table 3). 

**Table 3:** Serology testing of anti-MERS-CoV IgM and IgG in three close relatives travelling with the patient using MERS-CoV infected Vero cells for immunofluorescence assay, first imported MERS case in Thailand, June–July 2015

Close relatives at high-risk	First sera 19 Jun 2015 (day 5)	Second sera 1 Jul 2015 (day 17)
Relative 1, 45 years of age	Negative	Negative
Relative 2, 30 years of age	Negative	Negative
Relative 3, 25 years of age	Negative	Negative

MERS-CoV: Middle East respiratory syndrome coronavirus.

The Thai outbreak investigation identified 45 other high-risk close contacts, including 14 airline passengers who sat in the two rows around the index case’s seat, 12 airline crew members, 17 healthcare workers at the private hospital (first hospital of admission) in Bangkok and two taxi drivers. All but the 12 airline crew members were quarantined as the crew members left the country for their return flight operation on 15 June 2015 (day 1). However, once the diagnosis was confirmed, the crew members were notified and self-quarantined for 14 days. Laboratory testing carried out on samples collected from 36 high-risk close contacts all tested negative at least twice for MERS-CoV by real-time PCR. Further, none of the high-risk close contacts, including the airline crew members and healthcare workers, reported to have developed symptoms compatible with MERS-CoV infection during the quarantine period.

## Discussion

This study demonstrates the challenges faced by physicians and the cross-border threats that exist with increasing international medical tourism. Although precautions such as ThermoScan are in place at airports in light of the MERS-CoV outbreak in the Middle East and South Korea, cases can slip through checkpoints due to atypical presentations. The patient flew on a commercial airline despite his sickness and was not detected by the ThermoScan at the immigration checkpoint in Bangkok as he was afebrile. He only had a mild, non-productive cough. This case report also provides important lessons regarding clinical case identification. The clinical diagnosis was complicated due to the existence of congestive heart failure, a condition that predisposes to either community-acquired or nosocomial pneumonia of various aetiologies. Furthermore, initial chest radiographs did not show clear signs of interstitial pneumonia as expected with MERS-CoV infection. Various contact tracing methods involving the cooperation of several authorities and business institutes, such as the border control, airline, hotel management, traffic control, local authorities and hospitals, were used to track-down all potential contacts in order to prevent an outbreak. Phone calls, passenger manifests, surveillance videos and immigration cards were essential tools for the successful contact tracing.

Upper respiratory tract samples, such as nasopharyngeal and oropharyngeal, are often used to detect upper respiratory tract illnesses during the acute phase. In MERS-CoV infection, however, higher viral loads have been found in specimens from the lower respiratory tract [16], with sputum or endotracheal secretion samples yielding better results [17]. This aligns with the WHO interim guidelines, which encourage using lower respiratory tract samples if available [8]. Physicians, surveillance staff and laboratory personnel must be well-informed about the procedures, reliability and limitations of diagnostic tests, and should be able to recognise signs of mismatch between laboratory results and clinical presentations. In this case, the initial diagnostic testing of upper respiratory tract samples on days 2 and 3 caused a delay in diagnosis that could have facilitated onward transmission. Fortunately, the patient was isolated in a NPU upon initial admission; however, he could have exposed other patients and hospital workers during the 8 hours he spent in regular ICU after the initial false negative test results. The decision to conduct repeated laboratory testing, as well as to test lower respiratory tract samples, was driven by clinical assessment and knowledge of the virus excretion pattern reported in earlier cases.

The algorithm for diagnosing MERS in the current WHO interim guidelines for laboratory testing indicates that two positive real-time PCR tests are sufficient in diagnosing MERS, i.e. ORF-1a and UpE. However, we found that performing three simultaneous assays and sequencing for UpE, ORF-1a and RdRp genes in parallel, allowed for swift in-country confirmation of the presence of MERS-CoV and reporting within 24 hours, facilitating prompt outbreak control measures. There was no secondary transmission, not even to close relatives or the healthcare workers at BIDI where the patient was transferred after the diagnosis was confirmed, despite 170 contacts, including 48 high-risk close contacts [7].

Despite the successful outcome of infection control measures, the case provides an example of the risk of MERS-CoV infection importation. Aside from providing technical support through the MOPH Emergency Operations Centre, deployments of experts from the WHO South-East Asian Regional Office also greatly facilitated the exchange of information on possible modalities of MERS-CoV infection with the Omani MOH through the IHR (2005) mechanism [18]. This study therefore emphasises how important hospital and organisation collaboration, as well as cross-border cooperation, is to successful infection control in the event of an outbreak.
